# Translation and Linguistic Validation into Spanish of the Owner-Reported Outcome Measure “Helsinki Chronic Pain Index” (HCPI)

**DOI:** 10.3390/vetsci12090811

**Published:** 2025-08-26

**Authors:** María Olcoz, Miguel Ángel Cabezas, Ignacio A. Gómez de Segura

**Affiliations:** 1 Department of Animal Medicine and Surgery, University Complutense of Madrid, 28040 Madrid, Spain; dolorvet@gmail.com (M.Á.C.); ialvarez@ucm.es (I.A.G.d.S.); 2 Dolorvet Anestesia & Analgesia Veterinaria, C/Doce de Octubre 36, 28009 Madrid, Spain

**Keywords:** owner-reported outcome measure, osteoarthritis, pain assessment, chronic pain, dog, questionnaire, owner-reported outcome measure

## Abstract

Due to the challenges in evaluating chronic pain in dogs, owner-reported outcome measures have been developed, such as the Helsinki Chronic Pain Index (HCPI), originally written in Finnish and published in English, limiting its use among Spanish-speaking veterinarians and owners/caretakers. The goal of this study was to obtain an equivalent Spanish version of the HCPI. The translation process followed established guidelines. First, two native Spanish speakers independently translated the HCPI from English to Spanish. A veterinary professional and a third translator then compared the translations to create a unified version. Next, an independent linguist translated the reconciled Spanish version back into English. The research team, alongside one of the linguists, reviewed the translation to resolve any discrepancies. To conclude, a cognitive assessment was conducted with 62 dog owners from diverse demographics to evaluate clarity and understanding of the translated HCPI. This resulted in a linguistically validated Spanish version of the HCPI that is conceptually aligned with the original, as a first step to validate its use by Spanish-speaking veterinarians and researchers to manage chronic pain in dogs. The next step in the process is psychometric validation, which will ensure the tool’s reliability and applicability in both clinical and research settings.

## Simple Summary

The Helsinki Chronic Pain Index (HCPI) is an owner-reported outcome measure that assesses signs of chronic pain in dogs, such as that caused by osteoarthritis, based on their behavior and daily activities. Until now, this tool was available in several languages but not in Spanish, which limited its use among Spanish-speaking veterinarians and owners/caretakers. The aim of this study was to obtain a culturally and conceptually equivalent Spanish version of the HCPI. A linguistic validation process of the HCPI’s English version into Spanish was conducted following the latest published recommendations and guidelines. With the assistance of a team of bilingual English–Spanish translators, the translation process was carried out (direct translation, reconciliation, back-translation, and committee review), followed by a cognitive analysis with a group of 62 dog owners of different ages, genders, and socioeconomic backgrounds to make sure that all questions were clearly understood and culturally appropriate. To this end, a linguistically validated Spanish version of the HCPI was developed, as a first step toward enabling Spanish-speaking veterinarians, technicians, and caretakers to utilize it effectively.

## 1. Introduction

Pain assessment is fundamental to ensuring animal welfare, particularly for chronic conditions like osteoarthritis, which significantly impacts quality of life and is estimated to affect between 20% and 37% of dogs older than one year [1,2,3]. One of the main challenges in assessing the severity and extent of chronic pain in dogs lies in its subjective and complex nature [4], as well as its slow and intermittent progression, in which clinical signs are often subtle, gradual in onset, and very difficult for the veterinarian to evaluate in the clinic, as this setting only allows for a one-time assessment in an environment that is not familiar to the patient [5].

Over the years, various methods for assessing chronic pain, both objective and subjective, have been developed, each with its own advantages and disadvantages. Among objective methods, we can identify biological indices (heart and respiratory rates, blood pressure, pupillary dilation), measurement of biomarkers such as plasma cortisol levels (associated with joint inflammation) or cytokines, activity indices (pedometers and accelerometers), gait analysis (force platform/plate or pressure sensitive walkway gait analysis, etc.), nociceptive withdrawal reflexes, etc. More detailed information can be found regarding the different methods for assessing chronic pain in dogs and cats elsewhere [5]. However, they may be affected by data collection methods and other variables, such as pharmacological treatments, and they are often costly, requiring dedicated facilities, sophisticated equipment, and trained personnel for their operation.

Concerning the subjective methods, there has been increasing interest in behavior-based pain assessments conducted by the owners/caretakers, as this approach enables continuous and long-term evaluation within the animal’s normal environment, even though this has some limitations, including greater subjectivity, since observed behaviors may be influenced by both the surroundings and the individual performing the assessment. Among these tools, owner-reported outcome measures (OROM) have been developed [6]. These OROMs are derived from human medical equivalents that collect information directly from patients (symptoms, health-related quality of life or functional status) [6,7]. They consist of sequences of questions or items scored based on the observations or experiences of the respondent, with individual item scores used to calculate the overall OROM score [8].

Among available OROMs, the Helsinki Chronic Pain Index (HCPI) stands out for its comprehensive evaluation of chronic pain in dogs. The HCPI assesses 11 behavioral and activity parameters through simple, owner-friendly questions, with proven reliability, a multidimensional approach (evaluating mood, mobility, and activity levels), and practical applicability across diverse dog populations. It is a simple multifactorial descriptive pain questionnaire, leading to an index (0–44 points) by summing up scores for the 11 questions or items (0–4 points) regarding the dog’s mood, vocalization, willingness to move (at walk, trot, and gallop), willingness to play and jump, ease to lie down, rise, and move after a long rest, and move after a major activity. They belong to the same construct, that of chronic pain in dogs, and are easily applicable to all kinds of dogs, owners, and environments, aiming to assess chronic signs of pain caused by osteoarthritis in dogs.

The HCPI was initially developed in Finnish and validated at the University of Helsinki to assess dogs with chronic signs of pain caused by osteoarthritis [9,10]. It was later translated and validated into English [9] and also in Italian and Portuguese [11,12]. To improve reliability, the English version of the HCPI has been updated (HCPI-E2) based on customer feedback [8,13].

Linguistic validation, also referred to as cross-cultural adaptation, is the procedure used to evaluate the cultural relevance and conceptual equivalence of translated materials, ensuring that the content validity of the original version is preserved during translation [14]. This process minimizes the likelihood of invalid data caused by inaccurate translation and ensures that differences in responses across populations reflect genuine variations rather than inconsistencies arising from unsuitable data collection methods [4].

Despite its widespread use in other languages, and having been translated into English, Portuguese, and Italian, the absence of a Spanish version has limited the HCPI’s use in Spanish-speaking communities. Thus, the objective of this study was the translation and linguistic validation into Spanish as spoken in Spain (hereafter referred to simply as Spanish) of the most recent English version of the HCPI (HCPI-E2), following established cross-cultural adaptation protocols. Our goal was to produce a conceptually equivalent version that maintains the original’s clinical relevance while ensuring cultural appropriateness for Spanish-speaking dog owners and veterinarians. It was hypothesized that it would be possible to produce a Spanish translation that maintains both the cultural relevance and conceptual accuracy of the original English version.

## 2. Materials and Methods

Following authorization from the developer (Dr. Anna K. Hielm-Björkman), the translation of the HCPI-E2 (freely available for research purposes at https://dspace.uevora.pt/rdpc/bitstream/10174/19611/1/HCPI_E2.pdf, accessed on 26 November 2024) into Spanish was carried out. The guidelines and recommendations established by the World Health Organization (WHO), the International Society for Pharmacoeconomics and Outcomes Research (ISPOR), and others [14,15,16,17,18,19] were followed. The translators selected for this task had prior experience translating another OROM related to canine osteoarthritis. Given their subject matter expertise, detailed instructions on translation methodology were deemed unnecessary. Since the HCPI was published for the first time in English, directly translated from Finnish by the developers, and only the translated version can be found online, English was considered as the source language.

To begin the process, two native Spanish speakers independently translated the original English version into Spanish. One translator was a veterinary expert with technical knowledge and familiarity with the HCPI, while the other had no background in health sciences. Given that the HCPI is intended for pet owners who are not expected to have technical expertise, the inclusion of a non-veterinarian translator was a deliberate choice. After the initial translations were completed, a third Spanish linguist, along with a veterinary professional, reviewed and compared both versions. They worked together to resolve any differences and produce a single, harmonized translation (reconciliation). In the following phase, an independent linguist with native-level proficiency in English and fluency in Spanish carried out the back-translation, converting the unified Spanish version back into U.S. English. Afterward, the research team, together with one of the participating linguists, conducted a detailed review to identify and address any inconsistencies among the original English version, the Spanish translation, and the back-translated document. This step aimed to verify the accuracy of the translation and ensure that the wording and concepts were clearly conveyed. In the event that significant differences or difficulties in understanding the target language version were identified, a new translation process would be started. Once the translation was finalized, a thorough cognitive evaluation was conducted to assess the questionnaire’s clarity and relevance within the target population. While the WHO and ISPOR recommend a minimum of five participants per section—up to ten—for cognitive testing, this study included responses from 62 dog owners. These participants represented a diverse range of ages, genders, and socioeconomic backgrounds to ensure broad representation. Each participant was provided with a questionnaire that contained a concise explanation of the HCPI as well as an overview of the study’s objectives. They were instructed to carefully review the translated version of the HCPI, assess whether every question and corresponding response option was easily understood, and offer any suggestions for alternative wording or phrasing that could enhance the clarity and comprehensibility of the content. The only requirements for participating in the cognitive debriefing were that all respondents must have a perfect command of Spanish (both spoken and written) and be owners/caretakers of a dog, whether healthy or exhibiting signs of osteoarthritis.

The study protocol, including this questionnaire, received approval from the institutional ethics committee (Ref: CE_20230511-02_SAL, dated 11 May 2023). In the final stage, the research team carefully reviewed all participant feedback to determine whether any adjustments were needed, leading to the final version of the HCPI translation in Spanish.

## 3. Results

The process of translation and linguistic validation, along with the cognitive analysis, allowed for the development of a version of HCPI-E2 translated into Spanish that is conceptually equivalent to the English version (Supplementary Materials).

Translation

The two independent translations by native Spanish speakers were very similar, with 11 discrepancies between them. In the following phase of the process, a third linguist who is a native Spanish speaker carefully reviewed both existing versions. This expert analyzed and compared the two translations in detail, with the goal of merging them into a single, cohesive, and consistent version. During this unification, the linguist selected terms from either translation that were considered the most accurate and appropriate, considering the wording and intent of the original document. Additionally, when necessary, the linguist introduced alternative terms that more closely reflected the meaning and style of the source text to ensure that the final translation remained faithful to the original content. For example, in Question 4: “*The dog walks*:“, one of the linguists translated it as “*El perro pasea*:“ while the other translated it as “*El perro camina*:“, both concepts being synonymous. However, it was ultimately decided to use the term “*El perro pasea*:“ since the term “*caminar*” is generally associated with moving on foot, usually with a functional purpose, whereas “*pasear*” also implies walking but with a more relaxed, recreational intention. Another example is Question 5, “*The dog trots* (*moving diagonal limbs at the same time*, “*jogging*”):“ one of the linguists translated it as “*El perro trota* (*moviendo las extremidades al mismo tiempo*, “*correr*”)”, while the second translated it as: “*El perro trota* (*moviendo las patas diagonales al mismo tiempo*, “*correr*”)”. However, the reconciled version was: “*El perro trota* (*mueve las extremidades diagonales al mismo tiempo, corre a paso moderado*)”. Table S1 displays every difference found between the two separate translations as well as the final unified version that was created by combining them.

Throughout the back-translation stage, just 10 differences were identified when comparing the back-translated text to the original English version. The research team thoroughly reviewed all inconsistencies found between the original document, the combined Spanish translation, and the back-translated text to ensure accuracy and consistency across all versions. When discrepancies remained unresolved, the team consulted with the native English-speaking linguist involved in the process. Several of the identified differences were deemed insignificant, as they involved synonymous terms that could be used interchangeably without altering the meaning. For example, the term “*tutor*” was back-translated as “*caretaker*,” while in the original version, it was “*owner*.” Other examples were the answer option “*con mucha facilidad*,” translated as “*very easily*,” corresponding to the original term “*with great ease*”, and the sentence “*Marque solo una respuesta*—*la que mejor describa a su perro durante la semana anterior*” back-translated as “*Mark only one answer*—*the one that best describes your dog in the previous week*” referencing the original sentence “*Tick only one answer*—*the one that best describes your dog during the preceding week*”. In some instances, the back-translation was not an exact replica of the original version; however, the differences observed were very minor and did not change the overall meaning of the statement. As an example, in Question 5, the back-translation was “*The dog trots* (*moves the front and back diagonal legs at the same time, running at a moderate pace*)”, while the original document states “*The dog trots* (*moving diagonal limbs at the same time*; “*jogging*”)”. A similar situation occurred in Question 9: “*The dog rises from a lying position*:“ which was translated as “*El perro se levanta desde una posición tumbada*:“, and the back-translation was “*The dog stands/gets up from a sitting position*”.

After comparing the back-translation and the original version, no modifications were necessary. Table S2 shows all the differences between the original version and the back-translation.

Cognitive debriefing

Following the completion of the translation process, a total of 62 dog owners were surveyed to evaluate the clarity and readability of the Spanish version of the HCPI-E2 questionnaire. Participants were classified based on age, gender, and educational background. The group consisted of 30 men and 32 women. Age distribution was as follows: 11 participants were aged ≤ 29 years, 13 were between 30 and 39 years, 16 were between 40 and 49 years, 13 were between 50 and 59 years, and 9 were aged 60 or older. Educational levels included primary education (*n* = 6), secondary education (*n* = 5), high school (*n* = 18), and postgraduate education (*n* = 33).

The whole group of participants reported understanding the questionnaire items and response options without difficulty. However, five individuals proposed minor changes to certain phrases or words to enhance overall readability.

Three participants had difficulty with the answer option “*ni con facilidad*, *ni con dificultad*” in questions 4, 8, 9, 10, and 11. They considered the response somewhat ambiguous compared to the other options (“*con gran facilidad*”, “*con facilidad*”, “*con dificultad*”, and “*con gran dificultad*”), making it unlikely that anyone would choose this response. Some respondents suggested changing it to “*con cierta dificultad*”, which translates as “*with some difficulty*”. The authors decided not to make any changes, as the meaning of the question and the answers were understood, and the original version was faithfully reflected; the back-translation matched the original version, so that changing the answer options could affect the results of the questionnaire.

Three other participants had problems with the answer options “*ni alerta*, *ni indiferente*”, and “*indiferente*” in question 1 “*El estado de ánimo de su perro es*:“, considering them very similar and making it difficult to choose between them. Although these two options may be ambiguous and very similar, only three out of 62 participants had difficulties with this question. Since the back-translation was consistent with the original version, we decided not to alter the response in order to retain its original meaning.

Finally, one respondent suggested modifying question 11 “*El perro se mueve después de una actividad importante o ejercicio intenso*” to “*El perro se mueve después de una actividad fuerte o potente o ejercicio intenso*”. The authors finally decided to change it to “*El perro se mueve después de una gran actividad o ejercicio intenso*” since the term “*importante*” in Spanish usually refers to something that has importance, while the adjective “*gran*/*grande*” refers to something that exceeds in size and intensity what is common and regular; therefore, it is considered more appropriate to use this term.

At the end, following the cognitive debriefing, only one question was changed. Table S3 presents all feedback and recommendations from the cognitive analysis, along with the changes implemented.

Once a revised version was produced based on the previous results, a second cognitive analysis was not considered necessary.

## 4. Discussion

This study aimed to develop a tool originally developed in English for assessing chronic pain in dogs. To this end, a linguistically validated Spanish version of the HCPI was developed as a first step towards enabling Spanish-speaking veterinarians, technicians, and caretakers to utilize it effectively. The use of OROMs in veterinary practice is increasingly common. However, to standardize their application and ensure accessibility to all professionals and caretakers, translation and linguistic validation into different languages are essential. Since many of these tools were originally developed in English, their application in non-English-speaking regions, such as Spain and Latin America, is challenging. Notably, other OROMs, including the Canine Brief Pain Inventory and the Liverpool Osteoarthritis in Dogs, have already been translated into Spanish [20,21]. The HCPI itself was initially developed in Finnish, translated into English, and back-translated by independent, bilingual translators with medical expertise. However, the initial English version lacked psychometric validation, which necessitated its revision and update to the HCPI-E2.

For global implementation in clinical practice, and to ensure accessibility, translation and cross-cultural adaptation into other languages are mandatory. This process extends beyond simple translation, requiring adaptation and validation within the cultural context where the tool will be used [17]. Following established guidelines, this study adhered to recommended practices for cross-cultural adaptation [14,15,16,17,18,19,22], even though there is no universally agreed-upon method. For example, while two translators with medical expertise are typically suggested for independent direct translations, tools intended for non-professional users may benefit from one translator lacking technical knowledge. Similarly, recommendations for back-translations vary, ranging from one to two independent versions. Despite methodological discrepancies, the following essential steps remain consistent: direct translation, unification, back-translation, committee review, and cognitive debriefing.

The English HCPI was updated based on user feedback, particularly concerning mobility-related questions. Owners noted that their dogs “would” gladly perform actions but “could” not, highlighting a discrepancy in the questionnaire’s phrasing. Consequently, four of the eleven questions were reworded from “reluctantly/willingly” to “with difficulty/ease” [8,13]. This adjustment addressed gaps in the original English version, which, unlike the Finnish version, had not undergone psychometric validation. Given that the Spanish translation was based on this English version, psychometric analysis of the Spanish version becomes the next necessary step to ensure that its validity and reliability align with the original HCPI.

Overall, there were minimal differences between the direct translations, unified translation, back-translation, and the original document. This outcome was expected, as the HCPI is a relatively concise questionnaire with straightforward language designed for dog caretakers of diverse socioeconomic backgrounds without requiring veterinary expertise. Moreover, the translators involved in this study had prior experience translating similar OROMs, which helped maintain consistency and accuracy throughout the process.

Cognitive debriefing involved 62 surveys conducted with dog owners from various ages, genders, and socioeconomic backgrounds, ensuring that the target population was adequately represented. Current guidelines recommend reevaluating items if 20% or more of respondents find them unclear [18]. In this study, while some participants suggested improvements to certain items, less than 20% of respondents deemed the questionnaire unclear. Respondents primarily offered suggestions rather than indicating difficulties in comprehension. Consequently, no significant changes were required, except for one minor adjustment.

This study has several limitations. All participants were drawn from a single geographic region (Madrid province), excluding individuals from other parts of Spain, which may have introduced sociocultural biases into the results. Additionally, the translation was tailored to Spain’s cultural context; thus, modifications might be needed for broader application in other Spanish-speaking regions, such as Latin America and North America. Furthermore, the translators involved in the study did not hold official qualifications in linguistics or philology, which may have led to overlooked inconsistencies or grammatical errors. Including a philologist might have mitigated this risk. Nonetheless, the translators had experience in adapting similar OROMs, and the relatively simple language of the HCPI likely minimized the impact of this limitation.

Another limitation to consider in this work is that the translation of the HCPI into Spanish was not conducted from the original document in Finnish but rather from the latest English version. This could have led to small differences in the translation and to the Spanish version not corresponding exactly to the original version. However, if we look at other works such as those by Della Rocca et al. [11] and Matsubara et al. [12], where they translated and validated the HCPI into Italian and Portuguese, respectively, they used the English version as the source document, and in both cases, the findings confirmed that the Italian and Portuguese versions of the HCPI were valid, sensitive, reliable, and precise instruments for assessing dogs with osteoarthritis in countries where Italian and Portuguese are spoken. Therefore, if we extrapolate these results, there should not be relevant differences with the Spanish version. However, it should be reminded that, in this work, only the linguistic validation has been carried out, with the psychometric analysis still pending in order to fully validate this instrument.

The primary aim of cross-cultural validation is to guarantee that the translated instrument preserves the characteristics of the original and operates as intended [22]. Psychometric validation [18], often omitted in many guidelines for cross-cultural adaptation, is a critical step to ensure the tool’s clinical and research utility in different cultural settings.

While this study successfully achieved linguistic validation of the HCPI (E2 English version), the next step will involve psychometric validation of the Spanish version to confirm its reliability and validity, in the same way as was done for the Italian and Portuguese versions of the HCPI [11,12]. In other words, the process of translating and linguistically validating an OROM must be complemented by a comprehensive psychometric analysis. This analysis entails the systematic development and rigorous evaluation of an instrument specifically designed to measure complex and intangible constructs such as chronic pain [23]. Through this process, the instrument’s key measurement properties, including reliability, validity, responsiveness, and interpretability, among others, are thoroughly examined and established. In essence, linguistic validation represents the foundational initial phase that ensures that the translated scales maintain their clinical and research utility when adapted into different languages. Following this, psychometric validation serves as a subsequent step, providing empirical evidence that supports the instrument’s effectiveness and appropriateness for use within cultural environments that differ from those in which the original instrument was developed.

## 5. Conclusions

In conclusion, this work provides a linguistically validated Spanish version of the latest English available version of the HCPI (HCPI-E2). However, this work represents only the initial step toward the complete validation of the HCPI into Spanish. Psychometric validation of this translation will be the next step, ensuring the tool’s broader applicability and robustness in various clinical and research contexts and promoting its use among Spanish-speaking veterinarians and researchers for managing chronic pain in dogs.

## Data Availability

The Spanish translation of the English version of the HCPI (E2), Cognitive debriefing and informed consent and detailed information on the translation process can be found in Supplementary Materials.

## Supplementary Materials

The following supporting information can be downloaded at: https://www.mdpi.com/article/10.3390/vetsci12090811/s1, Spanish version of the HCPI (E2), Cognitive debriefing and informed consent, Table S1: Discrepancies between independent translations and unified translation); Table S2: Discrepancies between the original version and the back-translation and the modifications made); and Table S3: Cognitive debriefing and final review.

## Abbreviations

The following abbreviations are used in this manuscript:

HCPI	Helsinki Chronic Pain Index
OROM	Owner-Reported Outcome Measure
HCPI-E2	English version of the Helsinki Chronic Pain Index
WHO	World Health Organization
ISPOR	International Society for Pharmacoeconomics and Outcomes Research
