# Supplementary material for: Translation and Linguistic Validation into Spanish of the Owner-Reported Outcome Measure “Helsinki Chronic Pain Index” (HCPI)
Source: Vet Sci. 2025 Aug 26;12(9):811. doi: 10.3390/vetsci12090811 (PMC12474355; doi:10.3390/vetsci12090811)
Supplement: Supplementary file 1 [file vetsci-12-00811-s001.zip › Supp Mat 1. Translation - Spanish HCPI.pdf]

HELSINGIN YLIOPISTO
HELSING FORS UNIVERSITET
UNIVERSITY OF HELSINKI

CUESTIONARIO DEL TUTOR
HCPI-E2 - Índice de dolor crónico de Helsinki

Fecha__________ Cuestionario no. 1 2 3 4 5 6 7 8 9 10 _____________

Nombre del perro ________________________ Diagnóstico ________________________

Propietario/Tutor _______________________ Firma del propietario/tutor: ___________

Marque sólo una respuesta — la que mejor describa a su perro durante la semana anterior

1. El estado de ánimo de su perro es:

Muy alerta	Alerta	Ni alerta, ni indiferente	Indiferente	Muy indiferente
☐	☐	☐	☐	☐

2. El perro juega:

Muy dispuesto	Dispuesto	Reacio	Muy reacio	No juega en absoluto
☐	☐	☐	☐	☐

3. Valore la frecuencia con la que su perro vocaliza el dolor (quejas audibles, gemidos, gritos, etc.):

Nunca	Casi nunca	Algunas veces	A menudo	Muy a menudo
☐	☐	☐	☐	☐

4. El perro camina:

Con gran facilidad	Con facilidad	Ni con facilidad, ni con dificultad	Con dificultad	Con gran dificultad
☐	☐	☐	☐	☐

5. El perro trota (mueve las extremidades diagonales al mismo tiempo, corre a paso moderado):

Con gran facilidad	Con facilidad	Con cierta dificultad	Con gran dificultad	No trota en absoluto
☐	☐	☐	☐	☐

6. El perro galopa (corre a gran velocidad):

Con gran facilidad	Con facilidad	Con cierta dificultad	Con gran dificultad	No galopa en absoluto
☐	☐	☐	☐	☐

7. El perro salta (ej. al coche, al sofá…):

Con gran facilidad	Con facilidad	Con cierta dificultad	Con gran dificultad	No salta en absoluto
☐	☐	☐	☐	☐

8. El perro se tumba:

Con gran facilidad	Con facilidad	Ni con facilidad, ni con dificultad	Con dificultad	Con gran dificultad
☐	☐	☐	☐	☐

9. El perro se levanta desde una posición tumbada:

Con gran facilidad	Con facilidad	Ni con facilidad, ni con dificultad	Con dificultad	Con gran dificultad
☐	☐	☐	☐	☐

10. El perro se mueve después de un largo descanso:

Con gran facilidad	Con facilidad	Ni con facilidad, ni con dificultad	Con dificultad	Con gran dificultad
☐	☐	☐	☐	☐

11. El perro se mueve después de una gran actividad o ejercicio intenso:

Con gran facilidad	Con facilidad	Ni con facilidad, ni con dificultad	Con dificultad	Con gran dificultad
☐	☐	☐	☐	☐

¡Gracias por su ayuda!

Notas del veterinario:

____

____

____

____

____

Este índice de dolor crónico canino ha sido desarrollado por la Universidad de Helsinki, Finlandia.

Para obtener información sobre el HCPI, por favor póngase en contacto con Anna Hielm-Björkman, DVM, PhD en anna.hielm-bjorkman@helsinki.fi
