# Supplementary material for: Translation and Linguistic Validation into Spanish of the Owner-Reported Outcome Measure “Helsinki Chronic Pain Index” (HCPI)
Source: Vet Sci. 2025 Aug 26;12(9):811. doi: 10.3390/vetsci12090811 (PMC12474355; doi:10.3390/vetsci12090811)
Supplement: Supplementary file 1 [file vetsci-12-00811-s001.zip › Supp Mat 2. Tables S1, S2, S3.pdf]

Supplementary Material

Table S1. Discrepancies between independent translations and unified translation.

Original	Independent translation 1	Independent translation 2	Unified translation
1. The dog's mood *is:*	1. El estado de ánimo del perro:	1. El estado de ánimo de tu perro es:	1. El estado de ánimo de su perro es:
4. The dog walks:	4. El perro camina:	4. El perro pasea:	4. El perro camina:
With great ease	Con gran facilidad	Con mucha facilidad	Con gran facilidad
Neither with ease, nor with difficulty	Con alguna dificultad	Con algo de dificultad	Ni con facilidad, ni con dificultad
5. The dog trots (moving diagonal limbs at the same time; "jogging"):	5. El perro trota (moviendo las extremidades diagonales al mismo tiempo, "correr"):	5. El perro trota (moviendo las patas diagonales al mismo tiempo, "correr"):	5. El perro trota (mueve las extremidades diagonales al mismo tiempo, corre a paso moderado):
With some difficulty	Con alguna dificultad	Con algo de dificultad	Con cierta dificultad
With ease	Fácilmente	Con facilidad	Con facilidad
Neither with ease, nor with difficulty	Ni fácil ni difícil	Con algo de dificultad	Ni con facilidad, ni con dificultad
9. The dog rises from a lying position:	9. El perro se levanta desde una posición tumbada:	9. El perro se levanta cuando está tumbado:	9. El perro se levanta desde una posición tumbada:
10. The dog moves after a long rest:	10. El perro se mueve después de un largo descanso:	10. El perro se mueve después de un descanso largo:	10. El perro se mueve después de un largo descanso:
11. The dog moves after major activity or heavy exercise:	11. El perro se mueve tras una actividad o ejercicio intenso:	11. El perro se mueve después de una actividad importante o un ejercicio intenso:	11. El perro se mueve después de una actividad importante o ejercicio intenso:

Table S2. Discrepancies between the original version and the back-translation and the modifications made.

Original	Unified translation	Back-translation	Review by the research team and a native English-speaking linguist	Modifications made
Owner questionnaire: HCPI-E2	Cuestionario del tutor: HCPI-E2 – Índice de dolor crónico de Helsinki	*Caretaker's questionnaire: HCPI-E2 – Helsinki Chronic Pain Index/Score*	Although the back-translation is not exactly the same as the original version, no modification was considered, as there was no difference in the meaning of the question	None
"Tick only one answer – the one that best describes your dog during the preceding week"	"Marque sólo una respuesta – la que mejor describa a su perro durante la semana anterior"	*"Mark only one answer – the one that best describes your dog in the previous week"*		
1. The dog's mood is:	1. El estado de ánimo de su perro es:	*1. Your dog's attitude/mood is:*		
Very indifferent	Muy indiferente	*Reluctant*		
Reluctantly	Reacio	*Reluctant*		
3. Rate how often your dog vocalizes pain (audible complaining, whining, crying out, etc.):	3. Valore la frecuencia con la que su perro vocaliza el dolor (quejas audibles, gemidos, gritos, etc.):	*3. Evaluate the frequency with which your dog vocalizes pain (audible complaints, whines, barks (shouts), etc.):*		
With great ease	Con gran facilidad	*Very easily*		
With ease	Con facilidad	*Easily*		
Neither with ease, nor with difficulty	Ni con facilidad, ni con dificultad	*Neither easily nor with difficulty*		
5. The dog trots (moving diagonal limbs at the same time; "jogging"):	5. El perro trota (mueve las extremidades diagonales al mismo tiempo, corre a paso moderado):	*5. The dog trots (moves the front and back diagonal legs at the same time, running at a moderate pace):*		
Does not trot at all	No trota en absoluto	*Will not trot at all*		
6. The dog gallops (high speed running):	6. El perro galopa (corre a gran velocidad):	*6. The dog gallops (runs very fast/at full speed):*		
Does not gallop at all	No galopa en absoluto	*Will not gallop at all*		

Original	Unified translation	Back-translation	Review by the research team and a native English-speaking linguist	Modifications made
7. The dog jumps (eg. into car, onto sofa…):	7. El perro salta (por ejemplo, al coche, al sofá…):	*7. The dog jumps up (e.g. into the car, the sofa…):*		
Does not jump at all	No salta en absoluto	*Will not jump at all*		
9. The dog rises from a lying position:	9. El perro se levanta desde una posición tumbada	*9. The dog stands/gets up from a sitting position:*		
11. The dog moves after major activity or heavy exercise:	11. El perro se mueve después de una actividad importante o ejercicio intenso:	*11. The dog moves after important or intense exercise or activity:*		
This canine Chronic Pain Index has been developed at the University of Helsinki, Finland. For information about the HCPI, please contact Anna Hielm-Björkman, DVM, PhD at anna.hielm-bjorkman@helsinki.fi	Este índice de dolor crónico canino ha sido desarrollado por la Universidad de Helsinki, Finlandia. Para obtener información sobre el HCPI, por favor póngase en contacto con Anna Hielm-Björkman, DVM, PhD en anna.hielm-bjorkman@helsinki.fi	*This canine chronic pain indde has been developed by the Helsinki University of Finland. To obtain information on the HCPI, please contact Anna Hielm-Björkman, DVM, PhD at anna.hielm-bjorkman@helsinki.fi*		

Table S3. Cognitive debriefing and final review

Original	Unified translation	Cognitive debriefing	Modifications made	Second cognitive debriefing
Neither with ease, nor with difficulty	Ni con facilidad, ni con dificultad	Three respondents considered the response somewhat ambiguous, making it unlikely that anyone would choose this response. Some of them suggested changing it to "*con cierta dificultad*", which translates as "*with some difficulty*". It was decided not to make any changes, as the meaning of the question and the answers were understood, and the original version was faithfully reflected: the back-translation matched the original version, so that changing the answer options could affect the results of the questionnaire.	None	
Neither alert, nor indifferent	Ni alerta, ni indiferente	Three other participants had problems with the answer options "*ni alerta, ni indiferente*" and "*indiferente*" in question 1 "*El estado de ánimo de su perro es:*", considering them very similar and making it difficult to choose between them. Although they may be two ambiguous options with few differences between them, only 3 participants out of 62 had problems with this question, and as the back-translation matches the original version; in order to maintain the same meaning, it was decided to keep the answers and make no modifications.	None	
11. The dog moves after major activity or heavy exercise:	11. El perro se mueve después de una actividad importante o ejercicio intenso:	One respondent suggested modifying question 11 "*El perro se mueve después de una actividad importante o ejercicio intenso*" to "… *una actividad fuerte o potente o ejercicio intenso*". It was finally decided to change to "… *de una gran actividad o ejercicio intenso*" since the term "*importante*" in Spanish usually refers to something that has importance, while the adjective "*gran/grande*" refers to something that exceeds in size and intensity what is common and regular, therefore it is considered more appropriate to use this term.	11. El perro se mueve después de una gran actividad o ejercicio intenso:	It was not deemed necessary
