# Supplementary material for: Translation and Linguistic Validation into Spanish of the Owner-Reported Outcome Measure “Helsinki Chronic Pain Index” (HCPI)
Source: Vet Sci. 2025 Aug 26;12(9):811. doi: 10.3390/vetsci12090811 (PMC12474355; doi:10.3390/vetsci12090811)
Supplement: Supplementary file 1 [file vetsci-12-00811-s001.zip › Supp Mat 3. Cognitive debriefing and informed consent.pdf]

Encuesta de legibilidad de la versión española de la escala de valoración de dolor crónico "Índice de Dolor Crónico de Helsinki"

Todavía resulta difícil evaluar el grado de dolor crónico en los perros valorando su comportamiento dada nuestra dificultad para interpretar sus emociones. Para reducir la subjetividad de la valoración del dolor se han diseñado cuestionarios sencillos que son rellenados por el propietario o tutor de sus mascotas.

El cuestionario de valoración de dolor crónico denominado **Índice de Dolor Crónico de Helsinki** (**IDCH**; en inglés, *HCPI*, *Helsinki Chronic Pain Index*) es un cuestionario multifactorial descriptivo de 11 ítems o preguntas relacionadas con el estado de ánimo del perro, vocalizaciones, disposición para moverse y para jugar, etc., donde los propietarios/tutores marcan, en una escala descriptiva de 5 puntos (0 a 4), la respuesta que mejor describa el estado de su perro. La suma de las puntuaciones de las 11 preguntas proporciona un índice, que oscila entre 0 y 44, y que permite valorar el grado de dolor crónico del perro.

Sin embargo, el principal problema del **IDCH** es que ha sido desarrollado en finés y traducido a otros idiomas como el inglés o el italiano, pero no al español. Para facilitar su uso se ha elaborado una traducción al español, pero debe confirmarse que tiene el mismo significado y las frases son de fácil legibilidad para el propietario/tutor medio.

Para verificar dicha legibilidad hemos elaborado tres preguntas sencillas sobre cada pregunta del **IDCH** traducido al español, es decir, si la versión traducida es comprensible para el público en general. Es un formulario voluntario y totalmente anónimo. La información aquí obtenida será utilizada para la elaboración de un Proyecto de Investigación y la implantación de una herramienta que consideramos muy valiosa para mejorar la calidad de vida de los perros.

El formulario se divide en dos partes. La primera, con 3 preguntas de información demográfica básica y anónima. La segunda de valoración de cada una de las preguntas del **IDCH** para saber si se entiende correctamente o genera algún tipo de ambigüedad o dificultad en su lectura y comprensión y, cuando sea oportuno, se proporcione una alternativa que resulte más adecuada.

Si acepta participar en este proyecto y desea rellenar el formulario, por favor marque la casilla correspondiente a continuación:

☐ **Si acepto** ☐ **No acepto**

Muchas gracias por su colaboración.

El formulario comienza en el reverso de esta hoja.

Marque con una X el rango de edad al que pertenece

☐ 20-29 años ☐ 30- 39 años ☐ 40-49 años ☐ 50-59 años ☐ >60 años

Marque con una X el género con el que se identifica

☐ Hombre ☐ Mujer ☐ Ninguno de los anteriores

Marque con una X el nivel de estudios

☐ Primaria ☐ Secundaria ☐ Bachillerato ☐ Estudios universitarios

---

A continuación, se le van a mostrar uno a uno todos los elementos que componen el cuestionario **IDCH**, y seguidamente se formularán una serie de preguntas sobre el grado de comprensión de cada elemento:

CUESTIONARIO DEL TUTOR
HCPI-E2 - Índice de dolor crónico de Helsinki

Fecha__________ Cuestionario no. 1 2 3 4 5 6 7 8 9 10 _____________

Nombre del perro ________________________ Diagnóstico ________________________

Propietario/Tutor ________________________ Firma del propietario/tutor: ___________

¿Se entiende correctamente?

☐ Si ☐ No

En caso de no comprenderse o haber un término que no se entienda indíquelo a continuación:

En sus propias palabras, ¿lo redactaría de alguna otra forma de modo que le resultara más sencillo de leer o más comprensible? En ese caso indíquelo a continuación:

Marque sólo una respuesta — la que mejor describa a su perro durante la semana anterior

1. El estado de ánimo de su perro es:

Muy alerta	Alerta	Ni alerta, ni indiferente	Indiferente	Muy indiferente
☐	☐	☐	☐	☐

¿Se entiende correctamente la pregunta, así como las posibles opciones de respuesta?

☐ Si ☐ No

En caso de no comprenderse la pregunta o haber un término que no se entienda indíquelo a continuación:

En sus propias palabras, ¿redactaría de alguna otra forma la pregunta de modo que le resultara más sencillo de leer o más comprensible? En ese caso indíquelo a continuación:

Marque sólo una respuesta — la que mejor describa a su perro durante la semana anterior

2. El perro juega:

Muy dispuesto	Dispuesto	Reacio	Muy reacio	No juega en absoluto
☐	☐	☐	☐	☐

¿Se entiende correctamente la pregunta, así como las posibles opciones de respuesta?

☐ Si ☐ No

En caso de no comprenderse la pregunta o haber un término que no se entienda indíquelo a continuación:

En sus propias palabras, ¿redactaría de alguna otra forma la pregunta de modo que le resultara más sencillo de leer o más comprensible? En ese caso indíquelo a continuación:

Marque sólo una respuesta — la que mejor describa a su perro durante la semana anterior

3. Valore la frecuencia con la que su perro vocaliza el dolor (quejas audibles, gemidos, gritos, etc.):

Nunca	Casi nunca	Algunas veces	A menudo	Muy a menudo
☐	☐	☐	☐	☐

¿Se entiende correctamente la pregunta, así como las posibles opciones de respuesta?

☐ Si ☐ No

En caso de no comprenderse la pregunta o haber un término que no se entienda indíquelo a continuación:

En sus propias palabras, ¿redactaría de alguna otra forma la pregunta de modo que le resultara más sencillo de leer o más comprensible? En ese caso indíquelo a continuación:

Marque sólo una respuesta — la que mejor describa a su perro durante la semana anterior

4. El perro camina:

Con gran facilidad	Con facilidad	Ni con facilidad, ni con dificultad	Con dificultad	Con gran dificultad
☐	☐	☐	☐	☐

¿Se entiende correctamente la pregunta, así como las posibles opciones de respuesta?

☐ Si ☐ No

En caso de no comprenderse la pregunta o haber un término que no se entienda indíquelo a continuación:

En sus propias palabras, ¿redactaría de alguna otra forma la pregunta de modo que le resultara más sencillo de leer o más comprensible? En ese caso indíquelo a continuación:

Marque sólo una respuesta — la que mejor describa a su perro durante la semana anterior

5. El perro trota (mueve las extremidades diagonales al mismo tiempo, corre a paso moderado):

Con gran facilidad	Con facilidad	Con cierta dificultad	Con gran dificultad	No trota en absoluto
☐	☐	☐	☐	☐

¿Se entiende correctamente la pregunta, así como las posibles opciones de respuesta?

☐ Si ☐ No

En caso de no comprenderse la pregunta o haber un término que no se entienda indíquelo a continuación:

En sus propias palabras, ¿redactaría de alguna otra forma la pregunta de modo que le resultara más sencillo de leer o más comprensible? En ese caso indíquelo a continuación:

Marque sólo una respuesta — la que mejor describa a su perro durante la semana anterior

6. El perro galopa (corre a gran velocidad):

Con gran facilidad	Con facilidad	Con cierta dificultad	Con gran dificultad	No galopa en absoluto
☐	☐	☐	☐	☐

¿Se entiende correctamente la pregunta, así como las posibles opciones de respuesta?

☐ Si ☐ No

En caso de no comprenderse la pregunta o haber un término que no se entienda indíquelo a continuación:

En sus propias palabras, ¿redactaría de alguna otra forma la pregunta de modo que le resultara más sencillo de leer o más comprensible? En ese caso indíquelo a continuación:

Marque sólo una respuesta — la que mejor describa a su perro durante la semana anterior

7. El perro salta (ej. al coche, al sofá…):

Con gran facilidad	Con facilidad	Con cierta dificultad	Con gran dificultad	No salta en absoluto
☐	☐	☐	☐	☐

¿Se entiende correctamente la pregunta, así como las posibles opciones de respuesta?

☐ Si ☐ No

En caso de no comprenderse la pregunta o haber un término que no se entienda indíquelo a continuación:

En sus propias palabras, ¿redactaría de alguna otra forma la pregunta de modo que le resultara más sencillo de leer o más comprensible? En ese caso indíquelo a continuación:

Marque sólo una respuesta — la que mejor describa a su perro durante la semana anterior

8. El perro se tumba:

Con gran facilidad	Con facilidad	Ni con facilidad, ni con dificultad	Con dificultad	Con gran dificultad
☐	☐	☐	☐	☐

¿Se entiende correctamente la pregunta, así como las posibles opciones de respuesta?

☐ Si ☐ No

En caso de no comprenderse la pregunta o haber un término que no se entienda indíquelo a continuación:

En sus propias palabras, ¿redactaría de alguna otra forma la pregunta de modo que le resultara más sencillo de leer o más comprensible? En ese caso indíquelo a continuación:

Marque sólo una respuesta — la que mejor describa a su perro durante la semana anterior

9. El perro se levanta desde una posición tumbada:

Con gran facilidad	Con facilidad	Ni con facilidad, ni con dificultad	Con dificultad	Con gran dificultad
☐	☐	☐	☐	☐

¿Se entiende correctamente la pregunta, así como las posibles opciones de respuesta?

☐ Si ☐ No

En caso de no comprenderse la pregunta o haber un término que no se entienda indíquelo a continuación:

En sus propias palabras, ¿redactaría de alguna otra forma la pregunta de modo que le resultara más sencillo de leer o más comprensible? En ese caso indíquelo a continuación:

Marque sólo una respuesta — la que mejor describa a su perro durante la semana anterior

10. El perro se mueve después de un largo descanso:

Con gran facilidad	Con facilidad	Ni con facilidad, ni con dificultad	Con dificultad	Con gran dificultad
☐	☐	☐	☐	☐

¿Se entiende correctamente la pregunta, así como las posibles opciones de respuesta?

☐ Si ☐ No

En caso de no comprenderse la pregunta o haber un término que no se entienda indíquelo a continuación:

En sus propias palabras, ¿redactaría de alguna otra forma la pregunta de modo que le resultara más sencillo de leer o más comprensible? En ese caso indíquelo a continuación:

Marque sólo una respuesta — la que mejor describa a su perro durante la semana anterior

11. El perro se mueve después de una actividad importante o ejercicio intenso:

Con gran facilidad	Con facilidad	Ni con facilidad, ni con dificultad	Con dificultad	Con gran dificultad
☐	☐	☐	☐	☐

¡Gracias por su ayuda!

¿Se entiende correctamente la pregunta, así como las posibles opciones de respuesta?

☐ Si ☐ No

En caso de no comprenderse la pregunta o haber un término que no se entienda indíquelo a continuación:

En sus propias palabras, ¿redactaría de alguna otra forma la pregunta de modo que le resultara más sencillo de leer o más comprensible? En ese caso indíquelo a continuación:
